# Reproducibility of a single-volume dynamic CT myocardial blood flow measurement technique: validation in a swine model

**DOI:** 10.1186/s41747-024-00498-2

**Published:** 2024-08-14

**Authors:** Negin Hadjiabdolhamid, Yixiao Zhao, Logan Hubbard, Sabee Molloi

**Affiliations:** Department of Radiological Sciences, University of California, Irvine, Irvine, CA 92697 USA

**Keywords:** Computed tomography angiography, Coronary angiography, Coronary artery disease, Myocardial ischemia, Myocardial perfusion imaging

## Abstract

**Background:**

We prospectively assessed the reproducibility of a novel low-dose single-volume dynamic computed tomography (CT) myocardial blood flow measurement technique.

**Methods:**

Thirty-four pairs of measurements were made under rest and stress conditions in 13 swine (54.3 ± 12.3 kg). One or two acquisition pairs were acquired in each animal with a 10-min delay between each pair. Contrast (370 mgI/mL; 0.5 mL/kg) and a diluted contrast/saline chaser (0.5 mL/kg; 30:70 contrast/saline) were injected peripherally at 5 mL/s, followed by bolus tracking and acquisition of a single volume scan (100 kVp; 200 mA) with a 320-slice CT scanner. Bolus tracking and single volume scan data were used to derive perfusion in mL/min/g using a first-pass analysis model; the coronary perfusion territories of the left anterior descending (LAD), left circumflex (LCx), and right coronary artery (RCA) were automatically assigned using a previously validated minimum-cost path technique. The reproducibility of CT myocardial perfusion measurement within the LAD, LCx, RCA, and the whole myocardium was assessed via regression analysis. The average CT dose index (CTDI) of perfusion measurement was recorded.

**Results:**

The repeated first (P_myo1_) and second (P_myo2_) single-volume CT perfusion measurements were related by *P*_*myo2*_ = 1.01*P*_*myo1*_ − 0.03(ρ = 0.96; *RMSE* = 0.08 mL/min/g; *RMSE* = 0.07 mL/min/g) for the whole myocardium, and by *P*_*reg2*_ = 0.86*P*_*reg1*_ + 0.13(ρ = 0.87; *RMSE* = 0.31 mL/min/g; *RMSE* = 0.29 mL/min/g) for the LAD, LCx, and RCA perfusion territories. The average CTDI of the single-volume CT perfusion measurement was 10.5 mGy.

**Conclusion:**

The single-volume CT blood flow measurement technique provides reproducible low-dose myocardial perfusion measurement using only bolus tracking data and a single whole-heart volume scan.

**Relevance statement:**

The single-volume CT blood flow measurement technique is a noninvasive tool that reproducibly measures myocardial perfusion and provides coronary CT angiograms, allowing for simultaneous anatomic-physiologic assessment of myocardial ischemia.

**Key Points:**

- A low-dose single-volume dynamic CT myocardial blood flow measurement technique is reproducible.
- Motion misregistration artifacts are eliminated using a single-volume CT perfusion technique.
- This technique enables combined anatomic-physiologic assessment of coronary artery disease.

**Graphical Abstract:**

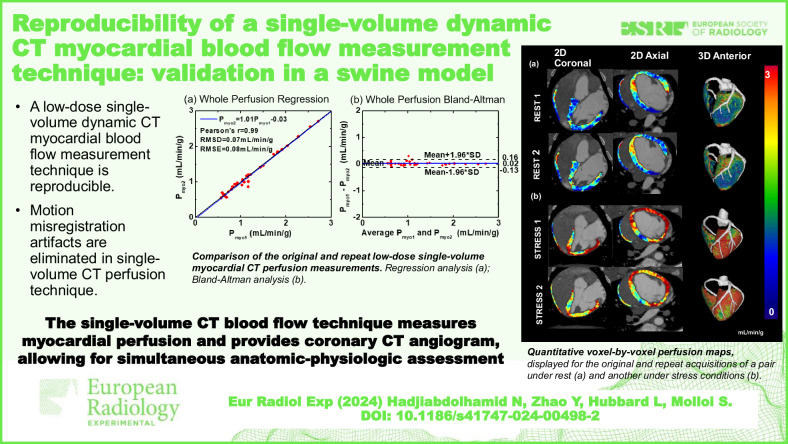

## Background

Coronary artery disease (CAD) is the leading cause of morbidity and mortality in the United States [1]. While coronary computed tomography angiography (CTA) is a powerful tool for anatomical assessment of CAD, there exist several approaches for evaluating its physiological effects. Dynamic computed tomography (CT) myocardial perfusion imaging, when used in conjunction with coronary CTA, improves diagnostic assessment of CAD by allowing for physiological assessment of ischemia [2–9]. Hence, dynamic CT perfusion offers an effective alternative to standard functional testing in patients with suspected CAD [10]. However, current CT perfusion techniques rely on multiple cardiac scans [5, 11, 12] to measure myocardial blood flow [13] resulting in high effective radiation doses of up to 10–15 mSv per exam [14, 15]. While several dose reduction strategies have been promising, such strategies still incur relatively high radiation exposures of approximately 5 mSv or greater [16]. Moreover, the need for multiple CT scans for perfusion measurement results in cardiac motion artifacts despite image registration.

Fortunately, a two-volume first-pass analysis dynamic CT perfusion technique was developed to decrease the radiation dose of perfusion measurement to less than 2 mSv [17, 18] and its accuracy was validated using fluorescent microspheres as the reference standard [19–21]. Specifically, two volume scans were acquired at the base (V1) and peak (V2) of aortic enhancement, where the delay time between V1 and V2 is determined using an optimal contrast injection protocol [17]. However, the clinical implementation of this technique can be limited by motion artifacts between the two volume scans [18]. A dynamic CT perfusion technique requiring only bolus tracking data and a single volume scan at the peak of contrast enhancement has recently been reported [19]. This technique minimizes the required radiation dose and eliminates motion misregistration artifacts between different volume scans. However, the reproducibility of this single-volume technique has yet to be established.

Hence, the purpose of this study was to assess the reproducibility of the single-volume quantitative CT myocardial blood flow measurement technique under rest and stress perfusion conditions. The central hypothesis was that the bolus tracking data combined with the acquisition of a single contrast-enhanced volume scan at the peak enhancement could reproducibly measure myocardial perfusion in mL/min/g at a low radiation dose.

## Methods

### General methods

This prospective study was approved by the Institutional Animal Care and Use Committee at the University of California Irvine (IACUC Protocol Number: AUP-22-015) and was carried out in accordance with all relevant regulations and guidelines. A total of 13 male Yorkshire swine (54.3 ± 12.3 ${{{\rm{kg}}}}$) were used in this validation study. Exclusion criteria were heart rate above 100 beats per minute, severe beam hardening, and false triggering caused by inappropriate ROI placement. The exclusion of datasets was based on one element from each couple presenting with exclusion criteria. Contrast-enhanced single-volume CT perfusion imaging with bolus tracking was performed under rest and stress conditions on each animal, as described below. The imaging protocol remained identical for all studies.

### Animal preparation

Anesthesia was induced with Telazol (4.4 mg/kg) and Xylazine (2.2 mg/kg) and was maintained through mechanical ventilation with 1.5–2.5% isoflurane (Highland Medical Equipment, Temecula, CA and Baxter, Deerfield, IL, USA). A sheath (7Fr, AVANTI®, Cordis Corporation, Miami Lakes, FL, USA) was placed in a femoral vein for intravenous fluid and contrast material administration. Another sheath was placed in a marginal ear vein for intravenous fluid and adenosine administration. At the end of each experiment, the animal was euthanized by KCl injection (1 mL/kg).

### CT imaging protocol

Contrast-enhanced single-volume CT perfusion imaging with bolus tracking was performed under rest and stress conditions on each animal, where reproducibility measurements were made by acquiring an average of one or two scan pairs at rest, followed by one or two scan pairs at stress. This resulted in a total of two to four scans per animal, all using the same protocol timing and CT settings. Specifically, for each rest or stress CT acquisition, 0.5 mL/kg of contrast material (Isovue 370, Bracco Diagnostics, Princeton, NJ, USA) was injected at 5 mL/s followed by a diluted contrast/saline chaser (0.5 mL/kg; 30:70 contrast/saline) at the same rate. The diluted chaser was used to optimize visualization of the right ventricular by providing sufficient opacification of the right ventricular blood pool while minimizing streak artifacts from highly attenuating contrast material [22]. Following contrast injection, dynamic bolus tracking was performed in the descending aorta with a triggering threshold set at 80 HU above the baseline blood pool HU. Following triggering, a delay time of one-half the contrast injection duration plus a 2-s dispersion delay was employed, after which a single whole-heart volume scan was acquired under electrocardiography-gating at approximately the peak of the aortic enhancement within the same breath hold [17]. Implementation of a prospective electrocardiography-gated technique ensured that image acquisition consistently took place during diastole, around 70% of the R-R interval, a targeted window commonly used for coronary CTA. Between acquisition pairs, a 10-min delay was employed to allow for adequate clearance and recirculation of contrast material. Of additional note, five minutes prior to each stress examination, intravenous adenosine was infused continuously (240 μg/kg/min, Model 55-2222, Harvard Apparatus, Holliston, MA, USA) through a marginal ear vein, and infusion was maintained until each stress image acquisition was complete. Last, a subset of three animals was also given sublingual nitroglycerin spray five minutes prior to each rest scan to maximally dilate the coronaries for optimal coronary CT angiogram.

All CT images were acquired at 100 kVp, 200 mA, rotation time of 0.35 s, 320 × 0.5 mm collimation, and field-of-view of 240 mm (Aquilion One, Canon Medical Systems, Tustin, CA, USA). All volume scans were reconstructed using an FC03 kernel, a voxel size of $0$.468 × 0.468 × 0.50 mm^3^, and an AIDR3D algorithm with a slice thickness of 0.5 mm. The CT dose index (CTDI) of the single-volume perfusion protocol was also recorded.

### CT perfusion measurement

The single-volume myocardial CT perfusion technique uses dynamic bolus tracking data and a single contrast-enhanced volume scan as analytical inputs into a novel first-pass analysis compartment model for quantitative perfusion measurement in mL/min/g [19]. In particular, the entire myocardium (left and right ventricular myocardium combined) was modeled as a single perfusion compartment. Assuming no contrast mass exits the myocardial compartment over the measurement duration, the average compartmental perfusion (P_ave_) is proportional to the first-pass rate of compartmental contrast mass accumulation $\left(\frac{{{{{\rm{dM}}}}}_{{{{\rm{c}}}}}}{{{{\rm{dt}}}}}\right)$, normalized by the aortic average input concentration (C_IN_) and total myocardial tissue mass (M_T_) [19–21].

First, the bolus tracking and volume scan data are used to reconstruct a full aortic contrast enhancement curve using a gamma variate fit [17]. The aortic average input concentration (C_IN_) is then derived by integrating the gamma variate fit curve (area under the fit curve) from the contrast arrival time to the volume scan acquisition time. Of note, the contrast arrival time is defined as when the gamma fit curve initially inflects up. The integration time is then defined as the time between the contrast arrival time and the single volume scan acquisition time at approximately the peak of aortic enhancement.

Second, the contrast mass accumulation within the entire myocardial compartment (M_c_) is calculated. The mass accumulation can be calculated between pre- and post-contrast volume scans for blood flow measurement. In the single-volume perfusion technique case, the first several non-enhanced bolus tracking data are used as pre-contrast data to estimate the pre-contrast myocardial tissue attenuation. The assumption is that the myocardial attenuation is spatially homogenous within the entire myocardium and can be obtained by averaging the myocardial tissue sample within a bolus tracking scan window. The average post-contrast myocardial enhancement is estimated using the contrast-enhanced volume scan at approximately the peak of aortic enhancement. Then, the whole compartment contrasts mass change (dM_C_) is derived by multiplying the average difference between pre- and post-contrast myocardial enhancement and the number of voxels of the entire myocardium. When divided by the integration time (dt), the rate of contrast mass accumulation $\left(\frac{{{{{\rm{dM}}}}}_{c}}{{{{\rm{dt}}}}}\right)$ is obtained.

Next, the myocardial tissue mass (M_T_, g) is calculated as the product of the number of voxels from the myocardium segmentation, the voxel volume (mL), and the myocardial density (g/mL). The average perfusion within the myocardial compartment (P_ave_, mL/min/g) can then be computed, as described in Eq. 1.

1 $${{{{\rm{P}}}}}_{{{{\rm{ave}}}}}={{{{\rm{M}}}}}_{{{{\rm{T}}}}}^{-1}{{{{\rm{C}}}}}_{{{{\rm{IN}}}}}^{-1}\frac{{{{\rm{d}}}}{{{{\rm{M}}}}}_{{{{\rm{c}}}}}}{{{{\rm{dt}}}}}$$

Last, to calculate the voxel-by-voxel perfusion, the per-voxel change in myocardial enhancement is estimated by subtracting the average pre-contrast myocardial attenuation from the contrast-enhanced volume scan on every single voxel. The per-voxel myocardial perfusion is then derived by normalizing the per-voxel enhancement change by the average myocardial enhancement, in mL/min/g.

### CT perfusion reproducibility

**Fig. 1:**
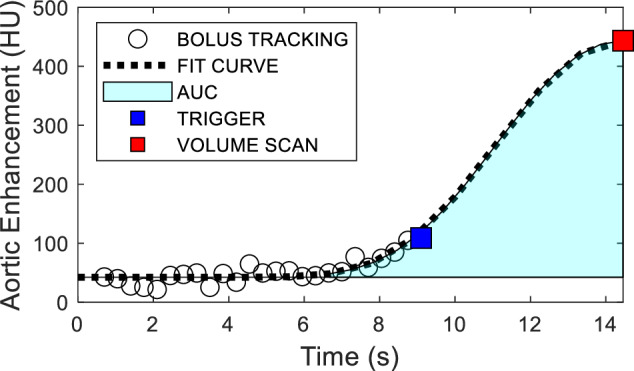
Example case of the single-volume quantitative CT perfusion acquisition protocol. Bolus tracking data points are aortic enhancement measurements from the descending aorta after contrast is injected until the arrival of contrast to the myocardial compartment. This is followed by triggering at 80 HU above the baseline HU. The volume scan is then acquired after a delay time of ½ injT + 2 s from the trigger. Aortic enhancement measurements from the bolus tracking and volume scan data were fit using automatic gamma variate fitting. Aortic-AIF, Dynamic aortic enhancement curve; AUC, Area-under-the-curve; FIT Curve, Gamma variate; Trigger, Aortic trigger; Volume Scan, Single-volume scan at the aortic enhancement peak

The reproducibility of the single-volume myocardial CT perfusion technique was assessed by comparing paired myocardial perfusion measurements under the same flow condition. For each acquisition, the central lumen of the descending aorta was segmented semi-automatically (Vitrea fX version 7.14.3.199, Vital Images, Inc., Minnetonka, MN, USA) from the volume scan to measure the peak aortic enhancement. The descending aorta within the 2-mm bolus tracking CT slices was also segmented to measure the aortic enhancement over time. The aortic enhancement obtained from the bolus tracking slices and volume scan were then fit automatically with the gamma variate function (LSQCurveFit, MatLab 2021, MathWorks, Natick, MA, USA), as shown in Fig. 1. Next, the entire myocardium, including both left and right ventricular myocardium, was semi-automatically segmented from the volume scan data. The myocardial tissue within the bolus tracking window was also segmented to estimate the pre-contrast myocardial tissue attenuation from the early bolus tracking CT slices. Perfusion measurements were then computed according to Eq. 1 using custom in-house software. In addition, semi-automatic extraction of the LAD, LCx, and RCA centerlines was performed. The vessel centerlines were then used by an automated minimum-cost path myocardial assignment technique to determine vessel-specific perfusion territories for each coronary artery by determining the minimum distance between each voxel of the myocardium and each coronary artery, then assigning each voxel to its nearest coronary artery [23, 24]. Regional perfusion measurements in the LAD, LCx, and RCA were then computed by averaging the per-voxel perfusion measurements within each assigned territory. After this, a comparison to their corresponding acquisition pair regional perfusion measurements was performed.

### Statistical analysis

Whole-myocardium perfusion measurements for each flow condition were compared to their corresponding acquisition pair perfusion measurement under rest and stress conditions. Whole-myocardium and vessel-specific regional perfusion measurements were compared pairwise with Wilcoxon signed-rank testing, regression, Bland-Altman with limits-of-agreement, Spearman correlation (ρ), root-mean-square-error (RMSE), and root-mean-square deviation (RMSD) analysis. The *p*-values lower than 0.050 were considered as significant. All analyses were conducted with statistical software, including Microsoft Excel (Microsoft Corporation, 2018) and SPSS (IBM Corporation, version 22).

## Results

### General

A total of 80 CT measurements were obtained at a heart rate of less than 100 beats per minute, with 12 measurements subsequently excluded, resulting in 34 paired (68 total) measurements for the reproducibility assessment. Exclusion criteria were severe beam hardening (8 cases), and false triggering caused by inappropriate ROI placement (4 cases). On average, two to three repeat pairs of measurements were obtained per animal. Among the included data, the average whole-heart rest and stress perfusion were 0.92 (0.71–1.20) mL/min/g and 1.17 (0.81–2.09) mL/min/g, respectively. The mean heart rate and mean arterial blood pressure of the swine were 80 bpm and 50 mmHg under rest, and 82 bpm and 67 mmHg under stress, respectively. The average time delay between bolus-tracking-based triggering and volume scan acquisition at approximately the peak of the aortic enhancement was 4.5 s.

### Qualitative reproducibility measurements

**Fig. 2:**
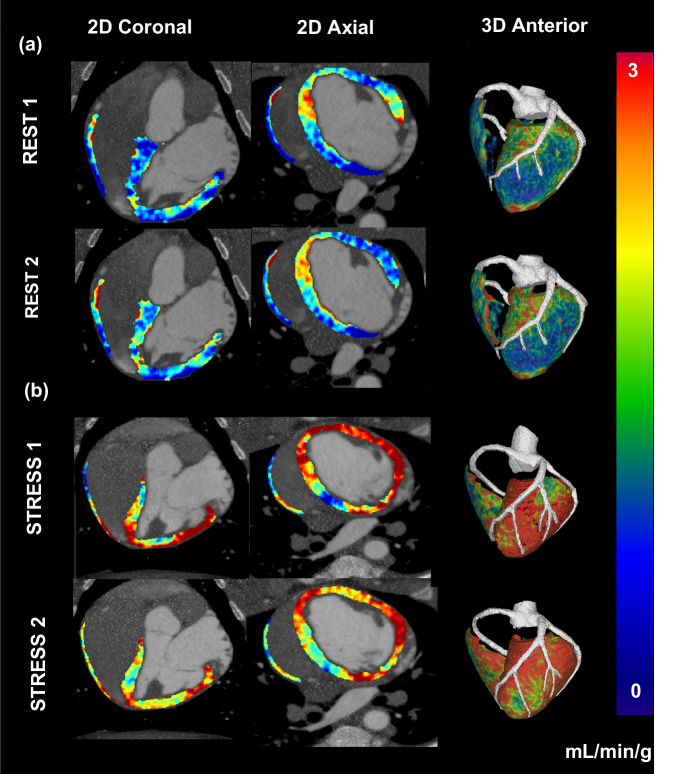
Quantitative voxel-by-voxel perfusion maps. The perfusion maps are displayed for the original and repeat acquisitions of a pair under rest (**a**) and another under stress conditions (**b**). The color bar indicates quantitative myocardial perfusion in mL/min/g

Representative coronal, axial, and 3D anterior perfusion maps of two paired acquisitions are shown under rest (Fig. 2a) and stress conditions (Fig. 2b) to visually compare the spatial distribution of the paired low-dose perfusion measurements.

### Quantitative reproducibility measurements

**Fig. 3:**
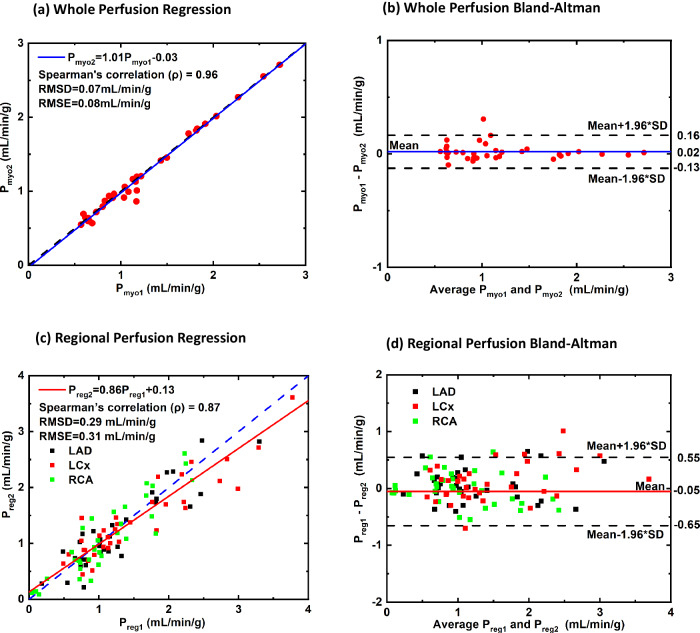
Comparison of the original and repeat low-dose single-volume myocardial CT perfusion measurements. A total of 68 perfusion measurements from 13 animals were assessed. Regression analysis comparing the original and repeat (**a**) whole-myocardium perfusion and (**c**) regional perfusion measurements of each acquisition pair. Corresponding Bland-Altman analysis for (**b**) whole perfusion and (**d**) regional perfusion are also displayed

The regression equation relating the first (${{{{\rm{P}}}}}_{{{{\rm{myo}}}}1}$) and second (${{{{\rm{P}}}}}_{{{{\rm{myo}}}}2}$) perfusion measurements in whole myocardium was ${{{{\rm{P}}}}}_{{{{\rm{myo}}}}2}=\,1.01{{{{\rm{P}}}}}_{{{{\rm{myo}}}}1}-0.03$ with a Spearman correlation (ρ) of 0.96, an RMSE of 0.08 mL/min/g and an RMSD of 0.07 mL/min/g (Fig. 3a). Similarly, paired perfusion measurements of all vessel-specific perfusion territories were linearly related by ${{{{\rm{P}}}}}_{{{{\rm{Reg}}}}2}=\,0.86{{{{\rm{P}}}}}_{{{{\rm{Reg}}}}1}+0.13$ with a Spearman correlation (ρ) of 0.87, a RMSE of 0.31 mL/min/g and a RMSD of 0.29 mL/min/g (Fig. 3c). The corresponding Bland-Altman analysis for whole myocardium and regional perfusions are shown in Fig. 3b, d, respectively. Under rest conditions, the median whole-myocardium perfusion for all first and second acquisitions were 0.91 (0.71–1.20) mL/min/g and 0.93 (0.71–1.20) mL/min/g, respectively (*p* = 0.876). Under stress conditions, the median whole-heart CT perfusion for all first and second acquisitions were 1.17 (0.86–2.15) mL/min/g and 1.16 (0.75–2.14) mL/min/g, respectively (*p* = 0.087). Detailed median whole-heart and vessel-specific perfusion measurements with their interquartile ranges are summarized in Table 1.

**Table 1:** Comparison of original and repeat perfusion measurements under rest and stress conditions

	Rest (mL/min/g)			Stress (mL/min/g)		
Region (*n* = 68)	Original, median (IQR)	Repeat, median (IQR)	*p*-value	Original, median (IQR)	Repeat, median (IQR)	*p*-value
Whole-heart	0.91 (0.48)	0.93 (0.49)	0.876	1.17 (1.29)	1.16 (1.39)	0.087
LAD	1.01 (0.61)	1.02 (0.57)	0.639	1.14 (1.13)	1.03 (1.05)	0.972
LCx	1.13 (0.99)	1.14 (0.82)	0.230	1.83 (1.56)	1.34 (1.42)	0.196
RCA	0.94 (0.51)	0.89 (0.72)	0.794	1.32 (1.31)	1.08 (1.60)	0.753

Data are expressed as median perfusion (IQR)

*IQR* Interquartile range, *LAD* Left coronary artery, *LCx* Left circumflex coronary artery, *n* Number of perfusion measurements, *RCA* Right coronary artery

Individual analysis for each perfusion territory was also performed, as shown in Table 2. For LAD, LCx, and RCA perfusion territories, paired perfusion measurements were related by ${{{{\rm{P}}}}}_{{{{\rm{LAD}}}}2}=\,0.88{{{{\rm{P}}}}}_{{{{\rm{LAD}}}}1}+0.10$, ${{{{\rm{P}}}}}_{{{{\rm{LCx}}}}2}=\,0.77{{{{\rm{P}}}}}_{{{{\rm{LCx}}}}1}+0.24$, and ${{{{\rm{P}}}}}_{{{{\rm{RCA}}}}2}=\,1.01{{{{\rm{P}}}}}_{{{{\rm{RCA}}}}1}-0.01$, respectively. The corresponding Spearman correlation (ρ), RMSE, and RMSD are specified in Table 2.

**Table 2:** Regression comparison of original and repeat perfusion measurements

Region (*n* = 68)	Slope	Intercept	Spearman correlation (ρ)	RMSE (mL/min/g)	RMSD (mL/min/g)
Whole-heart	1.01 [0.96, 1.05]	-0.03 [-0.09, 0.03]	0.96 [0.90, 0.98]	0.08	0.07
LAD	0.88 [0.73, 1.03]	0.1 [-0.11, 0.31]	0.81 [0.62, 0.90]	0.29	0.28
LCx	0.77 [0.65, 0.89]	0.24 [0.03, 0.45]	0.87 [0.68, 0.96]	0.35	0.28
RCA	1.01 [0.84, 1.17]	0 [-0.21, 0.20]	0.87 [0.67, 0.94]	0.28	0.28

For slope, intercept, and Spearman correlation (ρ), 95% confidence intervals (CI) are expressed as [CI_Lower_, CI_Upper_]

*LAD* Left coronary artery, *LCx* Left circumflex coronary artery, *n* Number of perfusion measurements, *RCA* Right coronary artery, *RMSE* Root-mean-square error, *RMSD* Root-mean-square deviation

**Fig. 4:**
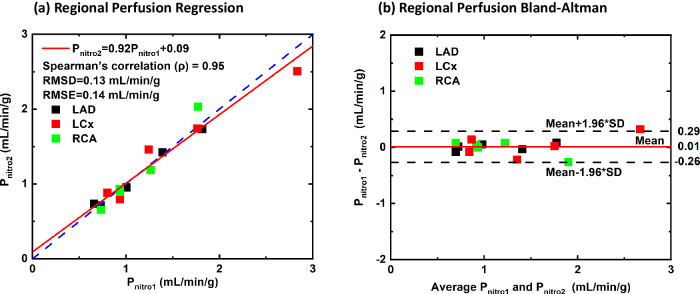
Comparison of the original and repeat low-dose single-volume myocardial rest perfusion measurements in vessel-specific perfusion territories after administration of sublingual nitroglycerine. A total of 10 perfusion measurements from a subset of 3 animals were assessed. **a** Regression analysis comparing the original and repeat regional perfusion measurements of each acquisition pair. **b** Corresponding Bland-Altman analysis for regional perfusion

For the subset of three animals that were given sublingual nitroglycerin under rest conditions, the regression equation relating the first and second perfusion measurements in the LAD, LCx, and RCA perfusion territories combined was ${{{{\rm{P}}}}}_{{{{\rm{Nitro}}}}2}=0.92{{{{\rm{P}}}}}_{{{{\rm{Nitro}}}}1}+0.09$ (ρ = 0.95, RMSE = 0.14 mL/min/g and RMSD = 0.13 mL/min/g, Fig. 4a). The corresponding Bland-Altman analysis is displayed in Fig. 4b.

### Radiation dose

The CTDI and dose-linear product of the single-volume CT perfusion measurement were 10.5 mGy and 168 mGy.cm, respectively. According to AAPM report No. 204 [25], the SSDE was calculated to be 13.6 mGy. By using a chest conversion factor of 0.014 mSv*mGy^−1^*cm^−1^ [25], the equivalent effective radiation dose in the patient was estimated to be 2.3 mSv.

## Discussion

### Indications from results

This study showed that the single-volume dynamic CT myocardial perfusion technique can reproducibly measure myocardial perfusion in mL/min/g in the whole myocardium with near unity regression slope, minimal RMSE and RMSD, and excellent Spearman correlation. While the results indicate that vessel-specific perfusion is less precise than whole-heart perfusion, the administration of sublingual nitroglycerine before coronary CT angiogram improved vessel-specific perfusion measurement. This is because a CT angiogram with more extensive and consistent coronary artery branches reduces the reproducibility error in myocardial mass assignment [23, 24].

The single-volume CT myocardial perfusion technique has several advantages over existing dynamic CT perfusion techniques. First, the single-volume technique enables quantitative perfusion measurement using bolus tracking data and a single first-pass contrast-enhanced volume scan, whereas current dynamic CT perfusions require 10–15 consecutively acquired image volumes for perfusion measurement. As such, the radiation dose associated with the single-volume technique is reduced as compared to current dynamic perfusion techniques. Second, the compartment modeling used by the single-volume technique improves the accuracy of perfusion measurement as compared to many current dynamic CT perfusion techniques that are known to underestimate perfusion, as previously shown [19]. Third, the single-volume technique minimizes respiratory motion artifact as only a short breath-hold time is necessary to acquire the peak enhancement volume scan, similar to a standard coronary CTA, as compared to existing perfusion techniques which require a breath-hold over approximately 10–15 cardiac cycles. Correspondingly, the single-volume technique also eliminates misregistration artifacts as compared to conventional dynamic CT perfusion techniques that require image registration algorithms to correct motion artifacts between volume scans prior to perfusion measurement. Fourth, the use of a diluted chaser improves visualization of the right ventricular myocardium, which in turn enables right ventricular myocardial perfusion measurement. Last, the single-volume perfusion technique’s acquisition protocol parallels that of a standard coronary CTA protocol; hence, diagnostic quality angiography data can be acquired simultaneously with perfusion data, assuming imaging is performed under resting conditions with a diagnostic tube current.

### Comparison to previous reports

Many research groups have aimed to reduce the radiation dose associated with dynamic CT perfusion. Application of radiation dose reduction techniques such as tube voltage reduction, tube current modulation, and scan acquisition number reduction, have all been promising [16, 26, 27]. For instance, tube voltage reduction was able to reduce the effective radiation dose by 40% (from 10.7 to 6.1 mSv) with preserved image quality and myocardial perfusion assessment in patients with normal body mass [26]. Automated tube current modulation was also able to achieve a reduced effective radiation dose of 7.7 mSv [27]. While tube voltage reduction and tube current modulation are widely used dose reduction strategies [28, 29], they remain limited. Since it is necessary to maintain a balance between radiation dose and diagnostic image quality, tube voltage, and tube current must remain within an acceptable range determined by patient size. Therefore, there has been a growing interest in CT perfusion techniques that utilize other methods to reduce dose, such as acquisition number reduction.

That said, when reducing the number of volume scans for existing perfusion techniques, most groups reported inaccurate myocardial perfusion measurements [27, 30]. Conversely, the previously reported first-pass analysis technique used only two optimally timed volume scans for accurate perfusion measurement [17, 20, 21, 31]. A CTDI of 2.3 mGy was reported, which is approximately 0.5 mSv [18]. More recently, this first-pass analysis technique was further optimized, enabling accurate dynamic CT perfusion measurement using bolus tracking data and a single contrast-enhanced volume scan, as validated *versus* ultrasound flow probe and microsphere flow [19]. Hence, the current work validated the prospective reproducibility of this single-volume CT perfusion technique. In this study, both rest and stress scans were acquired at 200 mA such that diagnostic quality coronary CTA data could be acquired simultaneously, where the CTA data was necessary for perfusion territory assignment and regional perfusion measurement. However, it is important to note that the radiation dose for perfusion measurement can be further reduced at 50 mA, as previously validated [19], assuming a diagnostic quality coronary CTA is not required.

### Limitations

First, no beam hardening correction algorithms were used while beam hardening artifacts were noticeable, resulting in false hypo-enhanced regions in the myocardium. Hence, acquisitions with severe beam hardening were excluded. The presence of beam hardening on the myocardium and the potential changes in the location of the affected area on the myocardium could explain the loss of precision when assessing regional perfusion repeatability across paired acquisitions. However, reducing contrast concentration and rate of contrast injection can help with reducing beam hardening to some extent, and additional beam hardening correction algorithms can be employed to further reduce errors [32, 33]. Second, myocardial assignment variations may exist when assigning vessel-specific perfusion territories using the minimum-cost path technique [23, 24], resulting in larger variations in vessel-specific perfusion measurements. Since the accuracy of this assignment, technique is dependent on the extent of the coronary artery branches, correct perfusion territory assignments are not possible without adequate visualization of coronary branches. Hence, if the length of the visualized vessels and branches were different for repeated acquisitions, variations in the perfusion territory assignment occurred. To address the issue with the assignment, sublingual nitroglycerin was administered for a subset of three animals to maximally dilate the coronary arteries for a more optimal coronary CT angiogram under rest conditions. This resulted in improved branch visualizations and vessel-specific perfusion measurement agreement in each perfusion territory. Third, it is important to note that our study used healthy swine subjects, which limits the evaluation of reproducibility to scenarios where myocardial ischemia is absent. While this approach allowed us to investigate the technique’s feasibility and initial performance metrics, future studies should aim to validate its efficacy in a myocardial ischemia model to assess the technique in more clinically relevant conditions. Fourth, regarding the stress perfusion rates assessed in the animals, several of the animals did not achieve an adequate response to adenosine, as evidenced by a non-significant difference between rest and stress heart rates. While this did not impact the accuracy of our results, it did reduce the dynamic range over which our measurements were assessed; hence future studies should employ higher adenosine concentrations or use regadenoson for improved hyperemic response. Last, the single-volume myocardial CT perfusion assumes that the myocardial attenuation is spatially homogenous within the entire myocardium. However, this assumption does not apply to patients with a prior history of conditions such as myocardial infarction or inflammatory cardiomyopathy. Both rest and stress perfusion measurements could be underestimated because of significant scarring. Alternatively, coronary flow capacity could be used to distinguish between scar and ischemia in such cases [34].

## Conclusions

The single-volume quantitative CT perfusion technique provides low-dose, reproducible myocardial perfusion measurement in mL/min/g using only bolus tracking data and a single whole-heart volume scan.

## Data Availability

The datasets used and/or analyzed during the current study are available from the corresponding author on reasonable request.

## Abbreviations

CAD: Coronary artery disease
CT: Computed tomography
CTA: CT angiography
LAD: Left anterior descending artery
LCx: Left circumflex artery
RCA: Right coronary artery
RMSD: Root-mean-square deviation
RMSE: Root-mean-square error
